# Change in Association Between Minerals, Fatty Acids and Oxidative Stress Parameters in Wistar Rats with Different Diets

**DOI:** 10.3390/ani16172678

**Published:** 2026-08-26

**Authors:** Slavica Ranković, Tamara Popović, Nevena Vidović, Saša Janković, Mirjana Lukić, Nikola Prvulović, Jasmina Debeljak Martačić

**Affiliations:** 1 Institute for Medical Research, National Institute of Republic of Serbia, University of Belgrade, Dr Subotića 4, 11000 Belgrade, Serbia; poptam@gmail.com (T.P.); nevenakardum@gmail.com (N.V.); nikola.prvulovic@imi.bg.ac.rs (N.P.); jasmina.martacic@imi.bg.ac.rs (J.D.M.); 2 Institute of Meat Hygiene and Technology, Kaćanskog 13, 11040 Belgrade, Serbia; sasa.jankovic@inmes.rs (S.J.); mirjana.lukic@inmes.rs (M.L.)

**Keywords:** Wistar rats, minerals, fatty acids, oxidative stress, desaturase and elongase indices, diet, regression

## Abstract

The liver plays a central role in fatty acid (FA) metabolism, triacylglycerol synthesis, and energy homeostasis. The study aims to evaluate how a four-week dietary intervention with fish-based (EF), milk-based (EM), and standard diets (ES) affects relationships between mineral levels, FA profiles and indices, and oxidative stress parameters in female Wistar rats. Liver tissue samples were collected and analyzed for these variables. Post hoc analysis revealed significant differences in trace elements between the EM and EF and the EM and ES groups, and in toxic trace elements only between the EM and EF groups. Individual mineral differences were observed for calcium (Ca), iron (Fe), copper (Cu), zinc (Zn), arsenic (As), mercury (Hg), and lead (Pb), all showing large effect sizes (ηp^2^ = 0.32–0.63). The strongest mineral–FA correlations occurred mainly in the ES group (15/25) and, to a lesser extent, in EF (9/25), involving 14 minerals, while only one mineral was significant in EM. Individual mineral correlations were most frequent in ES (13/25), followed by EF (8/25) and EM (4/25). Zn was the strongest predictor of malondialdehyde (MDA) in EF (90.6%), and Cu was the main determinant in ES (72.9%). We concluded that EM showed minimal interactions, limited to Fe and oleic acid, whereas EF and ES displayed multiple strong mineral–FA correlations, with ES exhibiting the most extensive and complex interaction network.

## Simple Summary

This study examines how different diets affect female Wistar rats and the relationships between mineral levels, fatty acid composition, and oxidative stress, which is a process that can damage cells and contribute to disease. Our results showed that rats fed a standard diet and a fish-based diet had stronger interactions between minerals and fatty acids than those fed a milk-based diet. The standard diet produced the greatest number of relationships, suggesting a more complex balance between nutrients involved in liver metabolism. We also found that specific minerals, including zinc, copper, and iron, were the strongest predictors of changes in oxidative stress, depending on the type of diet. These findings highlight the importance of diet composition in regulating how minerals and fatty acids interact to maintain normal liver function and antioxidant defense. This research helps us better understand how different dietary patterns influence metabolic health and may contribute to the development of nutritional strategies aimed at reducing oxidative stress and supporting liver health. The study also provides new insights into the complex interactions between minerals, fatty acids, and oxidative stress, which may be valuable for future animal nutrition research and potentially for improving human dietary recommendations.

## 1. Introduction

The liver is the primary organ for the metabolism of lipids and serves as the center for fatty acid (FA) synthesis and lipid export through lipoprotein production [1]. Minerals are essential dietary components involved in various physiological functions. They could be considered essential, as they are involved in establishing hormonal balance, serve as cofactors for enzyme activities, support optimal immunological response, and function as other biologically active molecules as well [2]. Macrominerals occur in higher proportions in body tissues and therefore are needed in larger dietary amounts. Microminerals are equally vital but required in smaller quantities and are essential trace elements, or oligoelements, which are needed only in very small daily amounts [3]. Rodents and mammals contain a range of mineral elements in their livers, which are essential for their numerous physiological functions. Livers consistently contain calcium (Ca), magnesium (Mg), sodium (Na), potassium (K), and phosphorus (P), then iron (Fe), zinc (Zn), copper (Cu), and manganese (Mn). Minerals are considered essential components with important roles in rat metabolic activity. Some enzymes’ functions include minerals in different metabolic pathways [4]. Mineral levels are generally constantly distributed throughout different regions of the liver. However, their presence can be affected by diet, sex, or some changes in organism conditions. For example, rats display alterations in liver FA composition due to exposure to diseases and toxins [5,6]. Looking at the association between minerals and FAs in diet, it can be noticed that some have a positive and some a negative direction; for example, Fe metabolism is negatively affected by polyunsaturated FAs (PUFAs) compared to saturated FAs (SFAs) [7]. Minerals and FAs frequently co-occur in various foods, mostly healthy ones like pulses [8]. They are closely connected through their synergistic roles in nutrition and dietary sources, forming complexes that enable stable structures. A good example is Zn, which functions as a cofactor of enzymes involved in FA metabolism [9]. Most Ca is found in teeth and bones, and its dietary mineral intake affects the absorption and function of fats. Mg, Zn, and selenium (Se) act together with (PUFAs) like docosahexaenoic acid (DHA) and eicosapentaenoic acid (EPA) to support neurogenesis and neurotransmitter synthesis [10]. Mg is also required for protein synthesis, as it stabilizes ribosomes [11,12]. Minerals and some products of FAs act as essential cofactors; precisely these non-protein components are important for enzyme activity, which enables numerous biochemical reactions, involving changes in metabolism, energy production, and cognitive function. Minerals generally function as inorganic enzymatic activators, while specific FA derivatives are involved in energy extraction. Thus, serum levels of PUFAs are associated with the serum levels of Zn and Mg [13]. Since mammals cannot synthesize linoleic acid (LA, 18:2 n-6) and alpha-linolenic acid (ALA, 18:3 n-3), these FAs must be obtained from the diet and are considered essential. Therefore, essential FAs must be present in the diet to generate some of the physiologically most important long-chain PUFAs [14]. FAs from membrane lipids have important metabolic, functional, and signaling roles.

The transformation of FAs in the liver depends on the activity of desaturases and elongases, key enzymes that introduce double bonds at specific positions along the carbon chain and extend the length of FAs, respectively. Depending on the number of C atoms as well as unsaturated bonds, FAs are divided into SFAs, monounsaturated FAs (MUFAs), and PUFAs. Their activity is vital for the metabolism of n-3 and n-6 PUFAs. The optimal ratio between total n-6 and n-3 PUFAs in the diet is important for health. n-3 FAs lower triglyceride levels, decrease inflammation, have immunomodulatory functions, and affect many aspects of cardiovascular function, including peripheral artery disease, major coronary events, and coagulation [15,16]. It is well known that PUFAs have regulatory effects on oxidative and inflammatory processes. A higher omega-3 (n-3) index is sex-specific, and in adult females it represents a predictor of lower malondialdehyde (MDA) concentrations. On the contrary, omega-6 FAs are inversely correlated with MDA [17].

Oxidative stress is usually defined as “a disturbance in the balance between the formation of reactive oxygen species (ROS) on the one hand and the activity of the antioxidant protection system (AOS) on the other hand” [18]. Antioxidant enzymes, including superoxide dismutase (SOD), catalase (CAT), glutathione peroxidases (GPx), and glutathione reductase, as well as antioxidant molecules present in plasma and cells, play a role in reducing intracellular ROS levels. SOD catalyzes the dismutation of superoxide anion radicals into hydrogen peroxide (H_2_O_2_), a less reactive species. Subsequently, CAT and GPx enzymes convert H_2_O_2_ to water and O_2_, completing the detoxification cascade [19]. Oxidative stress plays a pivotal role in various pathological conditions, such as hypertension, pulmonary hypertension, and diabetes, with high levels of oxidative stress in target organs. Oxidative stress is known to activate multiple intracellular signaling pathways, resulting in apoptosis or cell overgrowth, which can cause organ dysfunction. This indicates that targeting oxidative stress could be effective in the prevention of organ damage, and measuring oxidative stress status could serve as a biomarker in different disease states [20,21].

Although previous studies have described associations and potential mechanisms linking minerals and FAs, the combined and individual contributions of these nutrients, as well as their relationships with oxidative stress parameters under different dietary conditions, remain insufficiently investigated. A novel aspect of the present study is its multidimensional approach, integrating the assessment of mineral levels, FA profiles, desaturase activity, and oxidative stress parameters within the same experimental framework and across different dietary conditions. Furthermore, this study investigates which specific minerals, FAs, and metabolic indices are most strongly associated with oxidative stress parameters and whether these associations differ according to dietary condition. Therefore, this study aims to evaluate how relationships between mineral levels, FA profiles and indices, and oxidative stress parameters are altered in female rats following a four-week intervention with three distinct experimental diets: fish-based, milk-based, and standard diets.

## 2. Materials and Methods

### 2.1. Experimental Design and Treatment

The experiments were conducted on four-month-old female Wistar rats obtained from the vivarium of the Institute for Biological Research, University of Belgrade. The animals were housed under controlled conditions, including a room temperature of 23–25 °C, a 12 h light–dark cycle, and ad libitum access to food and drinking water.

All experimental procedures and animal maintenance protocols were performed in accordance with the Official Institutional Guide for Experimental Work on Animals, harmonized with the European Communities Council Directive (86/609) and the Guide for the Care and Use of Laboratory Animals (NIH publication no. 85–23). Approval for the study was granted by the Ethics Committee for the Use of Laboratory Animals of the Institute for Biological Research, University of Belgrade, under decision no. 02-56/12.

The animals were randomly divided into three treatment groups and were comparable in body weight (250–300 g). The control group (n = 6) received standard laboratory chow. In contrast, the fish-based group (n = 5) was provided with chow supplemented with fishmeal (anchovy-based), and the milk-based group (n = 8) received chow supplemented with dried milk powder during the 4-week treatment period.

The rat chow formulations were prepared by the Department of Animal Nutrition and Botany, Faculty of Veterinary Medicine, University of Belgrade. The main protein sources in the chow were either 9% fishmeal (anchovy) or 10% dried milk powder, with 16% and 15% soybean meal, respectively. All three diet types, fish-based, milk-based, and standard diets, had the following mineral composition (Ca, P, Na, Mg, Fe, and Zn). A comprehensive description of the rat chow composition and analytical dietary analysis is available in the previously published article [22].

FA methyl ester derivatives formed from isolated liver phospholipid fractions were separated by Gas Chromatography using a Shimadzu GC 2014 equipped with a flame ionization detector and an RTX 2330 fused silica gel capillary column. Individual FA methyl esters were identified by comparing sample peak retention times with authentic standards (Sigma Chemical Company, St. Louis, MO, USA) and/or PUFA-2 standard mixtures (Restek) [22].

### 2.2. Evaluation of Oxidative Stress Parameters

The preparation of tissue homogenates and the determination of TBARS levels, as well as SOD, GPx and CAT activities, were described in a previously published article [23]. Briefly, oxidative stress parameters were determined from previously prepared liver homogenates. All results were expressed per mg of protein. The concentration of TBARS, as a by-product of lipid peroxidation, was measured using a TBARS Assay Kit (Cayman Chemical, Ann Arbor, MI, USA) based on the reaction with TBA in an acidic pH at 90–100 °C. The SOD activity was determined using a Ransod kit (Randox, Crumlin, UK) based on superoxide radical anion production in a xanthine–xanthine oxidase system and its further reaction with 2-(4-iodophenyl)-3-(4-nitrophenol) phenyltetrazolium chloride, resulting in the formation of red formazan dye. The GPx activity was measured using a Ransel kit (Randox, Crumlin, UK) based on the oxidation of reduced glutathione in the presence of cumene hydroperoxide. CAT activity was determined by a slightly modified method described by Aebi [24], based on the degradation of hydrogen peroxide (H_2_O_2_ (Sigma-Aldrich^®^, Darmstadt, Germany), a reaction that can be measured directly by the decrease in absorbance at 240 nm. The protein content in tissue homogenates was determined using the bicinchoninic acid (BCA) Protein Assay Macro Kit (SERVA Electrophoresis, Heidelberg, Germany), according to the manufacturer’s instructions.

### 2.3. Evaluation of Minerals

Samples were kept frozen at −18 °C until analyzed. Approximately 0.5 g of homogenized sample was weighed on an analytical balance with an accuracy of ±0.001 g into the microwave digestion system Teflon vessel. Nitric acid (67% Trace Metal Grade; Fisher Scientific, Bishop, UK) and deionized water (0.063 μS) obtained from a water purification system (Purelab DV35; ELGA, Buckinghamshire, UK), were added to the sample in quantities of 5 mL each. The microwave digestion system (MARS 6; CEM Corporation, Matthews, NC, USA) was programmed as follows: 5 min from initial temperature to 180 °C, hold at 180 °C for another 10 min, cooling and venting for 20 min. Digested samples were quantitatively transferred into 100 mL polypropylene volumetric flasks and diluted with deionized water (0.063 μS).

Quantitative analysis of fifteen elements, Fe, Zn, Cu, Se, Mn, Cr, Na, K, Mg, Ca, P, Cd, Pb, Hg, and As, was performed by inductively coupled plasma mass spectrometry (ICP-MS). The iCap Q instrument (Thermo Scientific, Bremen, Germany) was used for analysis.

Optimization of plasma settings (torch position) and voltage parameters of the ion optics and electron multiplier was performed daily using tuning solution (Tune B, Thermo Scientific), provided by the instrument manufacturer to obtain the highest instrument response and sensitivity. Basic operating parameters were as follows: RF power (1550 W); cooling gas flow (14 L/min); nebulizer flow (1 L/min); collision gas flow (1 mL/min); dwell time (10 ms). All isotopes were measured in the KED (Kinetic Energy Discrimination) operating mode for elimination of interferences.

Individual calibration standards (1000 mg/L) of Fe, Zn, Cu, Se, Mn, Cr, Na, K, Mg, Ca, P, Cd, Pb and As were purchased from CPAchem (Bogomilovo, Bulgaria). Appropriate dilutions in 2% nitric acid were prepared to construct five-point calibration curves (including zero). A multielement internal standard consisting of ^6^Li, ^45^Sc, ^71^Ga, ^89^Y and ^209^Bi was used to improve accuracy and compensate for possible analyte losses.

An additional quality measure undertaken during analysis was assessment of certified reference material (NIST 1577c—bovine liver; Gaithersburg, MD, USA) in each analytical batch [25].

### 2.4. Statistical Analysis

Data were analyzed using SPSS Statistics for Windows, version 28.0 (IBM Corp., Armonk, NY, USA). To ensure privacy protection and reduce researcher bias, all data were entered using encrypted codes. Descriptive statistics, including arithmetic means and standard deviations, were calculated for all analyzed variables: grouped and individual minerals/elements, fatty acids (FAs), desaturase and elongase indices (INs), and oxidative stress parameters, including MDA, superoxide dismutase (SOD), glutathione peroxidase (GPx), and catalase (CAT).

Normality of distribution was assessed using the Kolmogorov–Smirnov test, which indicated suitability for parametric analysis. Differences between the dietary groups (milk-based group—EM, fish-based group—EF, and standard diet group—ES) for grouped and individual minerals/elements were evaluated using one-way analysis of variance (ANOVA), followed by the LSD post hoc test for pairwise comparisons.

Pearson correlation analysis was performed to examine associations between minerals/elements, FAs, and INs within each group. Correlation coefficients were interpreted as weak (r = 0.00–0.39), moderate (r = 0.40–0.69), or strong (r ≥ 0.70), according to [26]. Forward multiple regression analysis was used to identify the strongest predictors of oxidative stress parameters, separately adjusted for grouped minerals, individual minerals/elements, FAs, and desaturase/elongase indices. Statistical significance was set at *p* < 0.05.

## 3. Results

Table 1, Table 2 and Table 3 present descriptive statistics and tests of normality for groups of minerals, individual minerals, FAs, and the IN parameters. Across all three groups, only a small proportion of variables deviated significantly from a normal distribution based on the Kolmogorov–Smirnov test (*p* < 0.05), nine out of 46 in EM, five out of 46 in EF, and five out of 46 in ES. All variables were retained for parametric analyses due to the limited number of non-normally distributed variables, the consistent sample size across groups, and the established robustness of parametric methods to moderate deviations from normality.

**Figure 1:**
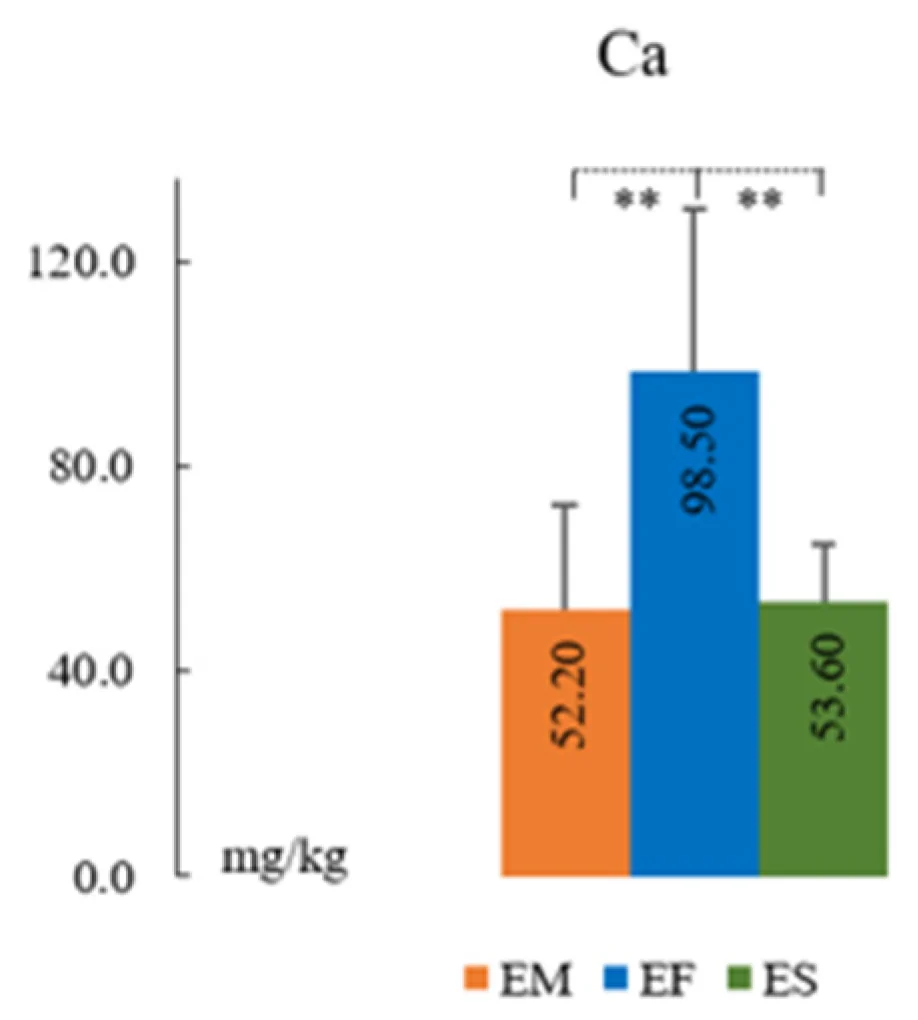
Differences between groups for individual macrominerals. Legends: **—statistical significance at *p* < 0.01.

**Figure 2:**
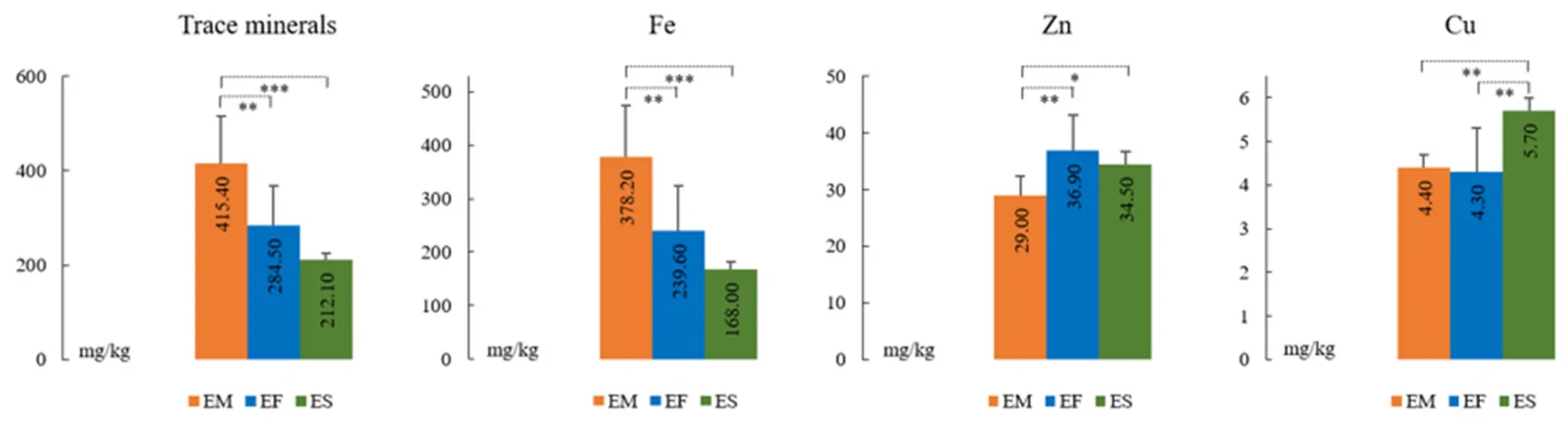
Differences between groups for group and individual trace minerals. Legends: ***—statistical significance at *p* < 0.001; **—statistical significance at *p* < 0.01; *—statistical significance at *p* < 0.05.

**Figure 3:**
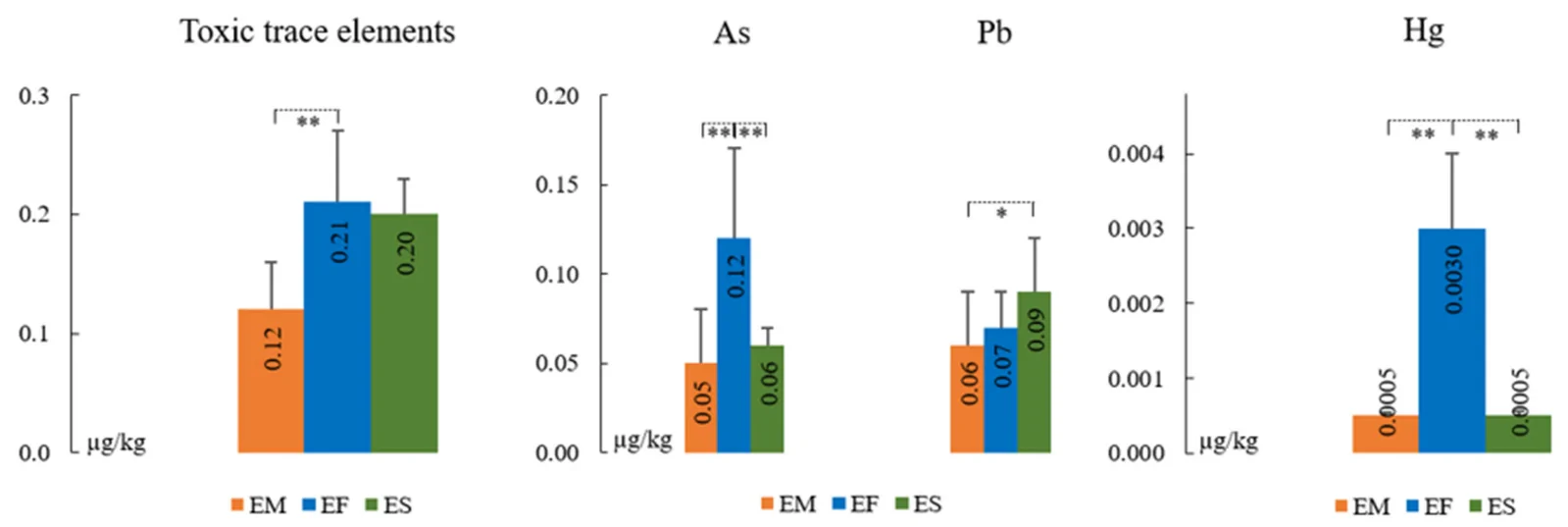
Differences between groups for group and individual toxic trace elements. Legends: **—statistical significance at *p* < 0.01; *—statistical significance at *p* < 0.05.

The post hoc LSD analysis revealed significant differences in trace elements between the EM and EF (mean difference = 130.9; 95% CI: 36.5–225.4) as well as between the EM and ES groups (mean difference = 203.3; 95% CI: 113.9–292.8). For toxic trace elements, significant differences were observed only between the EM and EF groups (mean difference = −0.09; 95% CI: −0.146 to −0.036). The analysis of individual minerals also indicated significant differences between groups for Ca, Fe, Cu, Zn, As, Hg, and Pb, all showing large effect sizes (ηp^2^ = 0.32–0.63). A detailed overview of between-group differences and post hoc comparisons, including the magnitude and direction of effects, is presented in Table 4 and Figure 1, Figure 2 and Figure 3.

Table 5 presents the correlations between mineral groups, individual elements, and FAs across dietary groups. Distinct patterns emerged depending on diet composition. Regarding individual minerals, the strongest positive and negative correlations were observed primarily in the ES (15 significant out of 25) and EF groups (9 significant out of 25), involving 14 different minerals and just one mineral in the EM group. In the ES group, seven correlations were strongly positive and eight strongly negative, whereas in the EF group, five were positive and four negative. Only one significant negative correlation was detected in the EM group, involving Fe. Several notable associations were identified between minerals and FAs, most prominently for Na, Cr, and Cd. Among the macrominerals, Na, Mg, and K exhibited multiple significant correlations with both monounsaturated fatty acids (MUFAs) and PUFAs.

Na showed multiple associations, positive with stearic acid (18:0) and negative with oleic acid (18:1n-9), vaccenic acid (18:1n-7), and linoleic acid (18:2) in the ES and EF groups. Mg correlated positively with vaccenic acid in EF and negatively with linoleic acid in ES, while K showed opposite trends: negative with stearic acid in ES and positive with vaccenic acid in EF. Ca was positively associated with adrenic acid (22:4) in ES.

Cr in ES demonstrated multiple links, positive with oleic and linoleic acids and negative with adrenic acid. Mn and Se correlated inversely and positively with arachidonic (20:4) and adrenic acid, respectively. Cu in EF showed a positive correlation with oleic acid and a negative one with arachidonic acid. Cd in ES exhibited both negative and positive correlations with several fatty acids, while Pb correlated negatively with stearic acid.

Table 6 presents the correlations between grouped and individual minerals and the calculated INs across dietary groups. Among the grouped minerals, two out of three categories showed significant relationships. Macrominerals exhibited a strong positive correlation with the delta 5 desaturase D5d (20:4/20:3) in the ES group, while trace minerals showed a strong negative correlation with the delta 9 desaturase D9d (18:1n-9/18:0) in the EM group. Toxic trace elements did not demonstrate any significant associations at the grouped level.

At the individual mineral level, significant correlations were most frequent in the ES group (13 out of 25), followed by EF (eight out of 25) and EM (four out of 25).

Within macrominerals, Mg showed two strong correlations, one positive with D5d (AA/DGLA) and one negative with delta 6 desaturase D6d (DGLA/LA), both in the ES group. Na and K each showed a single strong positive correlation with D9d (OA/SA) and D6d (DGLA/LA), respectively, while Ca had a positive correlation with the elongase Elo (AdA/AA) in ES.

Among trace elements, Cr displayed the highest number of associations, four in total, with three in ES (two positive: D9d OA/SA and Elo5 VA/PA; one negative: Elo AdA/AA) and one positive in EM (Elo5 VA/PA). Mn and Cu each showed two strong positive correlations in the EF and EM groups, respectively, while Fe and Se demonstrated one significant correlation each, negative in EM and positive in ES.

Within toxic elements, Cd showed the greatest number of associations (six in total). Three were strong positive correlations in ES with D9d (OA/SA), D9d (POA/PA), and Elo5 (VA/PA). In EF, Cd was negatively correlated with MUFAs and D9d (OA/SA), while in EM, it correlated positively with D6d (DGLA/LA). Four strong negative correlations, three in EF and one in ES, were mainly with desaturase and elongase indices.

Multiple regression analysis identified significant mineral predictors of oxidative stress markers across dietary groups. For MDA, Zn emerged as the strongest predictor in the EF group, explaining 91% of the unique variance. In the ES group, Cu initially accounted for 73% of the variance, while the addition of Fe contributed a further 26%. In the expanded models including Cu, Fe, and Cd, Cu and Fe retained strong independent effects, whereas Cd showed a smaller but still significant contribution.

For SOD activity, Ca was the only significant predictor in the ES group, explaining 89% of the unique variance, indicating a dominant role in antioxidant enzyme regulation within this dietary condition.

For GPx activity, Fe emerged as the primary determinant in the EM group, uniquely accounting for 77% of the variance. In contrast, no significant mineral or trace element predictors were identified for CAT activity in any dietary group.

Multiple regression analysis identified significant FA predictors of oxidative stress markers exclusively in the EM group. PA was a strong predictor of MDA, explaining 57% of its unique variance. For SOD, PA accounted for 76% of the variance, and the addition of arachidonic acid contributed another 19%. CAT was similarly influenced by PA, which explained 67% of its variance. In contrast, GPx activity was predicted solely by OA, uniquely accounting for 71% of the variance.

Multiple regression analysis identified significant desaturase, elongase, and FA index predictors of oxidative stress markers across the EM, ES, and EF groups. For MDA in the EM group, the n-6 PUFA index was the strongest predictor, explaining 71% of the unique variance. The addition of the D6d (20:3/18:2) index contributed a further 24–26% of the variance, while inclusion of the unsaturation index resulted in smaller but significant effects. In the ES group, D6d alone accounted for 72% of the variance in MDA, whereas the addition of the AA/EPA ratio contributed another 26%. For SOD activity, elongase activity Elo6 (18:0/16:0) was the primary predictor in the EM group, explaining 71% of the variance. In the EF group, D6d (20:3/18:2) was the dominant determinant, accounting for 96% of the unique variance. CAT activity was strongly predicted by the n-6 PUFA index in the EM and EF groups, explaining 76% and 95% of the variance, respectively. The addition of D6d provided smaller but independent contributions in both groups. For GPx activity in the EM group, the D9d (18:1n-9/18:0) index was the strongest predictor, uniquely explaining 75% of the variance, while D6d contributed an additional independent effect of 15%.

## 4. Discussion

The present study demonstrated that different dietary regimens altered the association patterns between minerals, FAs, desaturase indices, and oxidative stress parameters in Wistar rats. Distinct diet-dependent relationships were identified across experimental groups, suggesting that nutritional composition substantially modulates lipid metabolism, mineral homeostasis, and redox balance. Previous findings show that oxidative stress can directly impair FA oxidation and mitochondrial metabolic function, thereby influencing lipid remodeling and cellular homeostasis [27]. Omega-3 fatty acids and their derivatives are strongly anti-inflammatory, which dampens the metabolic effects of oxidative stress on the liver and peripheral tissues [28].

The ES and EF groups demonstrated a considerably higher number of significant correlations compared with the EM group, indicating that certain dietary patterns could promote stronger interactions between minerals and lipid metabolism (Table 5). The presence of both positive and negative correlations suggests that minerals exert differential regulatory effects depending on the FAs involved and the dietary environment. Among macrominerals, Na, Mg, and K showed the most significant correlations with MUFAs and PUFAs. The inverse relationships between Na and oleic, vaccenic, and linoleic acids, together with its positive association with stearic acid, reflect alterations in membrane lipid composition and oxidative processes associated with dietary fat quality. Excessive Na exposure has previously been linked with oxidative stress and inflammation capable of modifying lipid metabolism [29]. In contrast, Mg demonstrated favorable associations with unsaturated FAs, supporting its established role in lipid metabolism, insulin signaling, and antioxidant defense [30]. K also showed opposite trends with stearic and vaccenic acids, suggesting a possible modulatory role in membrane stability and cellular lipid regulation.

Looking at trace elements, Cr showed multiple correlations with FA profiles, including a positive correlation with oleic and linoleic acids. Cr is known to influence insulin sensitivity and glucose metabolism—pathways closely linked with FA synthesis and β-oxidation [31]. Another trace element, Se, had a positive correlation with adrenic acid, potentially reflecting its antioxidant role through selenoprotein-mediated protection against lipid peroxidation and maintenance of membrane integrity [32]. More evidence supports suggestions that selenoproteins exert synergistic protective effects against oxidative damage and lipid peroxidation under metabolic stress conditions [33,34]. Conversely, Mn and Cu showed inverse correlations with arachidonic acid, suggesting interactions with inflammatory and oxidative pathways. Cetin et al. [35] similarly demonstrated a close correlation between FA composition, antioxidant systems, and oxidative stress responses.

Significant results for toxic elements showed that Cd had both positive and negative correlations with several FAs, while Pb correlated negatively with stearic acid. Cd and Pb, as heavy metals, are recognized inducers of oxidative stress and lipid peroxidation that can disrupt membrane lipid composition and mitochondrial function [36]. Overall, these results support the concept that mineral–FA interactions are complex and strongly diet-dependent, potentially reflecting adaptive changes in oxidative and metabolic homeostasis.

Regarding correlations between minerals and desaturase and elongase indices, the ES group exhibited the highest number of significant results, suggesting that this dietary condition may cause greater modulation of the activity of both indices involved in lipid metabolism (Table 6). Previous studies have shown that dietary composition strongly affects FA desaturation pathways and membrane lipid remodeling under oxidative conditions [37,38]. Among grouped minerals, the positive correlations between macrominerals and D5d activity in the ES group reflect enhanced conversion of dihomo-γ-linolenic acid to arachidonic acid, potentially linked to altered inflammatory and oxidative pathways. Delta-5 desaturase activity is closely associated with inflammatory regulation and oxidative stress responses [39]. Conversely, the negative correlation between trace minerals and D9d activity in the EM group suggests a possible inhibitory influence on stearoyl-CoA desaturase activity and MUFA synthesis, processes known to be sensitive to micronutrient availability and metabolic stress [40].

At the individual level, Mg demonstrated strong relationships with both D5d and D6d indices, supporting its role as a cofactor in enzymatic lipid metabolism and antioxidant defense systems [30]. Na and K each correlated positively with the D9d index (OA/SA) or the D6d index (DGLA/LA). This association implies that electrolyte balance influences the D9d that generates monounsaturated fatty acids from saturated FAs and the D6d that initiates PUFA synthesis [13]. Trace elements (e.g., Cr, Mn, and Cu) exhibited multiple links with desaturase (D9d and D6d) and elongase (Elo5) indices, consistent with their known roles as cofactors for desaturase and elongase enzymes [13]. Additionally, Cr showed the greatest number of beneficial associations with desaturase and elongase indices, which reflects its involvement in insulin-mediated lipid regulation and oxidative balance [31]. In contrast, toxic elements, especially Cd and As, showed multiple correlations with altered desaturase and elongase activities, supporting the premise that heavy metals can disturb membrane lipid metabolism through oxidative-stress-mediated lipid peroxidation and mitochondrial dysfunction [41,42].

One of the regression analysis results showed associations between minerals and oxidative stress parameters, where Zn plays an important role in modulating oxidative balance as the strongest predictor of MDA in the EF group, while Cu and Fe showed substantial independent contributions in the ES group (Table 7). These findings are biologically plausible given the established involvement of Zn, Cu, and Fe in antioxidant defense and redox regulation. Oteiza [43] and Valko et al. [36] demonstrated that Zn stabilizes cell membranes and limits lipid peroxidation, while Cu and Fe participate in redox-sensitive enzymatic reactions and can have both antioxidant and pro-oxidant effects depending on concentration and metabolic conditions. The strong predictive results of Ca for SOD activity additionally support evidence that Ca signaling is closely linked to mitochondrial oxidative metabolism and antioxidant enzyme regulation [44].

Only significant FA predictors of oxidative stress markers were identified in the EM group, suggesting that this dietary condition benefits the coupling between lipid metabolism and redox homeostasis. PA was associated with MDA, SOD, and CAT, while OA predicted GPx activity (Table 8). SFAs, particularly PA, are known to promote oxidative stress through mitochondrial dysfunction and increased ROS production [45]. Findings in Listenberger et al. [46] showed that long-chain FAs induce oxidative stress-mediated cellular injury through mitochondrial and CD36-related pathways. In contrast, OA has protective effects by improving membrane stability and reducing lipid peroxidation in mice [47]. Although the dietary intervention in the study by Rudel et al. [48] lasted 16 weeks, which was longer than in our experiment, the authors demonstrated that OA modulates mitochondrial oxidative stress through GPx-related antioxidant mechanisms in mice. Palomino et al. demonstrated that pre- and co-treatment of cells with physiological concentrations of PA or OA conferred substantial protection of cell viability against an oxidative insult [49]. Another finding shows that arachidonic acid has interactions with inflammatory lipid mediators and antioxidant defense systems, which is in line with FA composition correlations with oxidative stress responses [35].

The results show that desaturase and elongase indices are also strong predictors of oxidative stress parameters, especially D6d and D9d activities (Table 9). In human experiments, indices are recognized as indirect markers of metabolic and inflammatory status [39]. The strong correlations between n-6 PUFAs, D6d activity, and oxidative stress parameters reflect increased susceptibility to lipid peroxidation under diets that promote higher unsaturated FA turnover. In a study by Mori et al. [50], results demonstrated that PUFAs both modulate and increase susceptibility to oxidative stress depending on dietary balance and antioxidant status. Similarly, the predictive value of Elo6 and D9d indices suggests that membrane remodeling and endogenous FA synthesis are closely connected with antioxidant responses.

Given the limited availability of comparable studies simultaneously examining minerals, FAs, desaturase activity, and oxidative stress across diverse dietary conditions, the present study provides additional insights into the complex metabolic relationships underlying the dietary modulation of redox homeostasis. This multidimensional approach represents a major strength of the study, contributing to a more comprehensive understanding of the potential interactions between mineral status, FA metabolism, and oxidative stress. Another limitation is that only female rats were included. This decision was made to maintain a sex-homogeneous experimental population and to minimize the potential confounding effects of sex-related differences in FA metabolism and desaturase activity. Previous studies have demonstrated sex differences in hepatic desaturase expression and long-chain PUFA metabolism, with female rats showing greater hepatic desaturase expression and, in some tissues, higher DHA concentrations than males [51]. Thus, while restricting the present study to females ensured a more homogeneous biological context for examining these metabolic relationships, it consequently limited the generalizability of the findings to males.

## 5. Conclusions

Based on the results, we concluded that the pattern of mineral and FA correlations differed markedly across diets. Animals fed the milk-based diet displayed a minimal interaction network, with only Fe negatively correlated with OA. In contrast, the fish-based diet produced multiple strong correlations between eight minerals and several FAs (palmitic, oleic, and arachidonic acids), while the standard diet showed the most extensive network, with nine minerals associated with five FAs (including saturated, monounsaturated, and polyunsaturated species). These findings suggest that dietary composition modulates the complexity of mineral–lipid interactions, with oleic acid emerging as a central FA across all groups. The denser correlation structure under standard and fish-based diets reflects greater involvement of minerals in FA metabolism and homeostasis, whereas the milk-based diet yielded more restricted associations. Additionally, across all models, oxidative stress markers showed distinct and diet-dependent predictor profiles. In the EM group, palmitic acid, oleic acid, n-6 PUFA indices, and elongase/desaturase activity were the strongest determinants of oxidative stress parameters. In contrast, the EF and ES groups were characterized by mineral- and enzyme-related predictors. Overall, oxidative stress regulation was highly group-specific and driven by different metabolic pathways, the underlying mechanisms of which remain to be fully clarified. Ultimately, this research enhances our understanding of how dietary patterns influence metabolic health, offering valuable insights for future animal nutrition studies and the development of human dietary recommendations aimed at mitigating oxidative stress. Future studies should include both sexes to determine whether the observed responses and relationships are sex dependent.

## Figures and Tables

**Table 1:** Descriptive statistics—minerals.

		Mean ± SD	
Var	EM	EF	ES
Macro	4469.7 ± 179.0 †	4458.1 ± 213.7	4449.2 ± 132.7
Trace	415.4 ± 99.1	284.5 ± 83.6	212.1 ± 14.1
Toxic	0.12 ± 0.04	0.21 ± 0.06	0.2 ± 0.03
Na	1204.9 ± 529.4 †	973.6 ± 187.6	1924.9 ± 175.3
Mg	218.4 ± 19.9 †	213.9 ± 22.6	236.6 ± 12.2
K	2994.1 ± 690.8 †	3172.0 ± 379.1 †	2866.1 ± 218.8
Ca	52.2 ± 20.3	98.5 ± 31.9	53.6 ± 11.1
Cr	0.3 ± 0.1	0.4 ± 0.1	0.3 ± 0.2
Mn	2.4 ± 0.2	2.2 ± 0.5	2.3 ± 0.1
Fe	378.2 ± 96.7	239.6 ± 84.7	168.0 ± 13.1
Cu	4.4 ± 0.3 †	4.3 ± 1.0	5.7 ± 0.3†
Zn	29.0 ± 3.3	36.9 ± 6.2	34.5 ± 2.2
Se	1.0 ± 0.3	0.8 ± 0.4	1.0 ± 0.1
As	0.05 ± 0.03	0.12 ± 0.05	0.06 ± 0.01
Cd	0.013 ± 0.01	0.017 ± 0.004	0.016 ± 0.01
Hg	0.0005 ± 0.00004	0.003 ± 0.002	0.0005 ± 0.00005
Pb	0.06 ± 0.03	0.07 ± 0.02	0.09 ± 0.02

Legends: Var—Variables; EM—Milk-based group; EF—Fish-based group; ES—Standard group; Macro—Macrominerals; Trace—Trace minerals; Toxic—Toxic trace elements; Na—Natrium; Mg—Magnesium; K—Kalium: Ca—Calcium; Cr—Chrome; Mn—Mangan; Fe—Iron; Cu—Copper; Zn—Zinc; Se—Selenium; As—Arsenic; Cd—Cadmium; Hg—Mercury; Pb—Lead. Note: † Variables not normally distributed (Kolmogorov–Smirnov test, *p* < 0.05).

**Table 2:** Descriptive statistics—fatty acids.

		Mean ± SD	
Var	EM	EF	ES
SFA	42.6 ± 1.0	45.3 ± 1.7	46.4 ± 5.2
MUFA	6.9 ± 0.6	6.0 ± 0.9	6.6 ± 1.1
PUFA	50.4 ± 1.2 †	48.7 ± 1.7	46.9 ± 4.9
n-3	10.1 ± 0.7	15.8 ± 1.9	8.6 ± 2.5
n-6	40.3 ± 1.5	32.8 ± 1.3	38.4 ± 3.5
n-6/n-3	4.0 ± 0.4 †	2.1 ± 0.3	4.8 ± 1.2
EPA + DHA	9.3 ± 0.6	14.6 ± 1.9	7.9 ± 2.3
AA/EPA	63.7 ± 11.8	12.0 ± 3.1	54.7 ± 7.6
EPA/AA	0.02 ± 0.004	0.09 ± 0.02	0.02 ± 0.003
D9d OA/SA	0.15 ± 0.01	0.12 ± 0.03 †	0.1 ± 0.02
D9d POA/PA	0.04 ± 0.02 †	0.04 ± 0.01 †	0.02 ± 0.01
D5d AA/DGLA	26.2 ± 3.9	16.0 ± 1.0	46.8 ± 22.7 †
D6d DGLA/LA	0.08 ± 0.02	0.1 ± 0.01	0.03 ± 0.01
Elo5 VA/POA	3.9 ± 1.2	3.3 ± 1.2	9.1 ± 6.3 †
Elo6 SA/PA	1.6 ± 0.2	1.7 ± 0.4	1.4 ± 0.2
Elo AdA/AA	0.02 ± 0.004	0.01 ± 0.003	0.01 ± 0.003
usat. Index	200.7 ± 3.6	203.5 ± 10.4	179.2 ± 22.6

Legends: SFA—Saturated fatty acid; MUFA—Monounsaturated fatty acid; PUFA—Polyunsaturated fatty acid; n-3—Omega-3 polyunsaturated fatty acid; n-6—Omega-6 polyunsaturated fatty acid; n-6/n-3—Omega-6 polyunsaturated fatty acid/omega-3 polyunsaturated fatty acid; EPA + DHA—Eicosapentaenoic acid + docosahexaenoic acid; AA/EPA—Arachidonic acid/eicosapentaenoic acid; EPA/AA—Eicosapentaenoic acid/arachidonic acid; D9d 18:1n-9/18:0—Desaturase index of oleic acid/stearic acid; D9d 16:1n-7/16:0—Desaturase index of palmitoleic acid/ palmitic acid; D5d 20:4n-6/20:3n-6—Desaturase index of arachidonic acid/dihomo-γ-linolenic acid; D6d 20:3n-6/18:2n-6—Desaturase index of dihomo-γ-linolenic acid/linoleic acid; Elo5 18:1n-7/16:1n-7—Elongase-5 activity index of vaccenic acid/palmitoleic acid; Elo6 18:0/16:0—Elongase-6 activity index of stearic acid/palmitic acid; Elo 22:4n-6/20:4n-6—Elongase activity index of adrenic acid/arachidonic acid. Note: † Variables not normally distributed (Kolmogorov–Smirnov test, *p* < 0.05).

**Table 3:** Descriptive statistics—indices and oxidative stress.

		Mean ± SD	
Var	EM	EF	ES
PA	16.3 ± 1.0	17.1 ± 2.7	19.7 ± 4.1
POA	0.68 ± 0.37 †	0.71 ± 0.35	0.47 ± 0.2
SA	26.3 ± 1.0	28.2 ± 1.7 †	26.6 ± 1.8
OA	3.9 ± 0.3	3.3 ± 0.8 †	2.7 ± 0.4 †
VA	2.3 ± 0.2	2.0 ± 0.2	3.5 ± 0.8
LA	12.9 ± 0.8	12.3 ± 1.2	15.4 ± 1.4
DGLA	1.0 ± 0.1	1.2 ± 0.1	0.5 ± 0.1
AA	25.9 ± 1.5	19.1 ± 0.5	22.3 ± 2.7 †
EPA	0.42 ± 0.1	1.67 ± 0.4	0.42 ± 0.1
AdA	0.39 ± 0.1	0.21 ± 0.1	0.18 ± 0.1
DPA 22:5	0.87 ± 0.1	1.27 ± 0.1	0.69 ± 0.3
DHA 22:6	8.8 ± 0.6	12.9 ± 1.6	7.5 ± 2.3
MDA	0.79 ± 0.26	1.1 ± 0.1	1.0 ± 0.08
SOD	12.1 ± 5.5	7.5 ± 2.5	15.6 ± 7.3
CAT	104.2 ± 37.5	147.2 ± 15.9	99.4 ± 10.0
GPx	1.72 ± 0.52	3.52 ± 0.73	1.9 ± 0.1

Legends: MDA—Malondialdehyde; SOD—Superoxide dismutase: GPx—Glutathione peroxidase; CAT—Catalase; 16:00—Palmitic acid (PA); 16:1n-7—Palmitoleic acid (POA); 18:0—Stearic acid (SA); 18:1n-9—Oleic acid (OA); 18:1n-7—Vaccenic acid (VA); 18:2n-6—Linoleic acid (LA); 20:3n-6—Dihomo-gamma-linolenic acid (DGLA); 20:4n-6—Arachidonic acid (AA); 20:5n-3—Eicosapentaenoic acid (EPA); 22:4n-6—Adrenic acid (AdA); 22:5n-3—Docosapentaenoic acid (DPA); 22:6n-3—Docosahexaenoic acid (DHA). Note: † Variables not normally distributed (Kolmogorov–Smirnov test, *p* < 0.05).

**Table 4:** Group and individual mineral/element differences between groups.

Var	F	Sig.	η_p_^2^	Post Hoc	Mean Diff 95%CI
Macro	0.024	0.977	0.003		
**Trace**	12.183	0.001	0.604	**EM-EF**	130.9 ** (36.5–225.4)
				**EM-ES**	203.3 *** (113.9–292.8)
				EF-ES	x
**Toxic**	6.389	0.009	0.444	**EM-EF**	−0.0912 ** (−0.146–−0.036)
				EM-ES	x
				EF-ES	x
Na	1.039	0.376	0.115	x	x
Mg	2.425	0.120	0.233	x	x
K	0.492	0.620	0.058	x	x
**Ca**	8.142	0.004	0.504	**EM-EF**	−46.3 ** (−72.6–−20.0)
				EM-ES	x
				**EF-ES**	44.9 ** (17.0–72.9)
Cr	1.693	0.215	0.175	x	x
Mn	0.635	0.543	0.074	x	x
**Fe**	13.469	0.000	0.627	**EM-EF**	138.6 ** (45.4–231.7)
				**EM-ES**	210.1 *** (121.9–298.4)
				EF-ES	x
**Cu**	11.173	0.001	0.583	EM-EF	x
				**EM-ES**	−1.3 ** (−1.9–−0.6)
				**EF-ES**	−1.4 ** (−2.1–−0.7)
**Zn**	6.882	0.007	0.462	**EM-EF**	−7.9 ** (−12.7–−3.1)
				**EM-ES**	−5.5 * (−10.1–−1.0)
				EF-ES	x
Se	1.246	0.314	0.135		x
**As**	8.694	0.003	0.521	**EM-EF**	−0.0749 ** (−0.11–−0.04)
				EM-ES	x
				**EF-ES**	0.05973 ** (0.018–0.101)
Cd	0.495	0.618	0.058		x
**Hg**	8.517	0.003	0.516	**EM-EF**	−0.00258 ** (−0.004–−0.001)
				EM-ES	x
				**EF-ES**	0.0025873 ** (0.001–0.004)
**Pb**	3.803	0.045	0.322	EM-EF	x
				**EM-ES**	−0.03182 * (−0.056–−0.007)
				EF-ES	x

Legends: η_p_^2^—partial eta squared; x—no significance; ***—statistical significance at *p* < 0.001; **—statistical significance at *p* < 0.01; *—statistical significance at *p* < 0.05. Note: In bold are significant results/variables.

**Table 5:** Correlation between groups of minerals/elements, individual minerals/elements and fatty acids for all groups.

Var	PA	SA	OA	VA	LA	DGLA	AA	AdA
Macro				(EF) 0.931 *		(ES) −0.909 *		
Trace			(EM) −0.717 *					
Toxic		(ES) −0.948 **						
Na		(ES) 0.851 *	(ES) −0.928 **	(EF) −0.993 **	(ES) −0.922 **			
Mg				(EF) 0.911 *		(ES) −0.913 *		
K		(ES) −0.828 *		(EF) 0.981 **				
Ca								(ES) 0.899 *
Cr			(ES) 0.930 **		(ES) 0.913 *			(ES) −0.902 *
Mn							(EF) −0.945 *	
Fe			(EM) −0.720 *					
Cu			(EF) 0.880 *				(EF) −0.916 *	
Zn						(ES) −0.829 *		
Se							(EF) −0.944 *	(ES) 0.875 *
As	(EF) 0.911 *							
Cd		(ES) −0.917 **	(ES) 0.914 *		(ES) 0.900 *		(EF) 0.947 *	
Pb		(ES) −0.830 *						

Legends: **—statistical significance at *p* < 0.01; *—statistical significance at *p* < 0.05. Note: Only Hg did not have any significant correlations with FAs, and FAs, POA, EPA, DPA, and DHA did not have any significant correlations with any of the minerals for all three groups.

**Table 6:** Correlations between groups of minerals/elements, individual minerals/elements, and desaturase and elongase indices for all groups.

Var	MUFA	PUFA	D9d OA/SA	D9d POA/PA	D5d AA/DGLA	D6d DGLA/LA	Elo5 VA/PA	Elo AdA/AA	Usat. Index
Macro					(ES) 0.878 *				
Trace			(EM) −0.741 *						
Na			(ES) −0.951 **						
Mg					(ES) 0.857 *	(ES) −0.833 *			
K						(ES) −0.920 **			
Ca								(ES) 0.825 *	
Cr			(ES) 0.924 **				(EM) 0.710 * (ES) 0.896 *	(ES) −0.855 *	
Mn	(EF) 0.919 *					(EM) 0.848 **			
Fe			(EM) −0.746 *						
Cu	(EF) 0.969 **		(EF) 0.955 *						
Se								(ES) 0.827 *	
As		(EF) −0.970 **			(EF) −0.932 *	(ES) −0.890 *			(EF) −0.879 *
Cd	(EF) −0.941 *		(EF) −0.883 * (ES) 0.966 **	(ES) −0.830 *		(EM) 0.815 *	(ES) 0.891 *		

Legends: **—statistical significance at *p* < 0.01; *—statistical significance at *p* < 0.05. Note: Toxic trace elements, Zn, Hg, and Pb, did not have any significant correlations with desaturase and elongase indices, and eight out of 17 desaturase and elongase indices, SFA, n-3, n-6, n-6/n-3, EPA + DHA, AA/EPA, EPA/AA, and Elo6 18:0/16:0, did not show any significant results with any of the minerals for all three groups.

**Table 7:** Multiple regression of independent associations between oxidative stress parameters and minerals/elements for all groups.

Var	G	Predictor	β	Adj. R^2^	Part r^2^ (%)	Sig.
MDA	EF	Zn	0.952	0.876	0.906 (90.6)	0.012
	ES	Cu	−0.854	0.661	0.729 (72.9)	0.031
		Cu, Fe	−0.701 −0.535	0.986	0.452 (45.2) 0.263 (26.3)	0.001 0.002
		Cu, Fe, Cd	−0.639 −0.562 −0.106	0.998	0.254 (25.4) 0.266 (26.6) 0.008 (0.8)	0.001 0.001 0.043
SOD	ES	Ca	0.941	0.857	0.885 (88.5)	0.005
CAT	x					
GPx	EM	Fe	0.878	0.732	0.770 (77.0)	0.004

Legends: β—Standardized; x—No significant results; Sig—Statistical significance.

**Table 8:** Multiple regression of independent associations between oxidative stress parameters and fatty acids for all groups.

Var	G	Predictor	β	Adj. R^2^	Part r^2^ (%)	Sig.
MDA	EM	16:00	−0.756	0.500	0.571 (57.1)	0.030
SOD	EM	16:00	−0.870	0.717	−0.757 (75.7)	0.005
		16:00 20:4n-6	−2.098 −1.302	0.923	−0.485 (48.5) −0.187 (18.7)	0.001 0.009
CAT	EM	16:00	−0.817	0.613	−0.667 (66.7)	0.013
GPx	EM	18:1n-9	−0.843	0.662	0.710 (71.0)	0.009

Legends: β—Standardized; x—No significant results; Sig—Statistical significance.

**Table 9:** Multiple regression of independent associations between oxidative stress parameters and desaturase and elongase indices for all groups.

Var	G	Predictor	β	Adj. R^2^	Part r^2^ (%)	Sig.
MDA	EM	n-6	0.842	0.660	0.709 (70.9)	0.009
		n-6 D6d 20:3/18:2	0.581 −0.552	0.924	0.262 (26.2) 0.237 (23.7)	0.004 0.006
		n-6 D6d 20:3/18:2 usat. ind	0.393 −0.634 0.254	0.969	0.069 (6.9) 0.274 (27.4) 0.037 (3.7)	0.016 0.001 0.044
	ES	D6d 20:3/18:2	0.847	0.648	0.718 (71.8)	0.033
		D6d 20:3/18:2 AA/EPA	0.825 −0.509	0.961	0.679 (67.9) 0.258 (25.8)	0.003 0.011
SOD	EM	Elo6 18:0/16:0	0.844	0.664	0.712 (71.2)	0.008
	EF	D6d 20:3/18:2	−0.977	0.940	0.955 (95.5)	0.004
CAT	EM	n-6	0.871	0.718	0.758 (75.8)	0.005
		n-6 D6d 20:3/18:2	0.657 −0.452	0.884	0.336 (33.6) 0.159 (15.9)	0.006 0.027
	EF	n-6	−0.973	0.928	0.946 (94.6)	0.005
		n-6 D6d 20:3/18:2	−0.790 0.294	0.999	0.384 (38.4) 0.053 (5.3)	0.001 0.007
GPx	EM	D9d 18:1n-9/18:0	−0.865	0.705	0.748 (74.8)	0.006
		D9d 18:1n-9/18:0 D6d 20:3/18:2	−0.752 −0.404	0.857	0.522 (52.2) 0.150 (15.0)	0.004 0.042

Legends: β—Standardized; x—No significant results; Sig—Statistical significance.

## Data Availability

The data presented in this study are available within the article.

## Abbreviations

The following abbreviations are used in this manuscript:

ALA	Alpha-linolenic acid, 18:3 n-3
AA	Arachidonic acid, 20:4 n-6
AdA	Adrenic acid, 22:4 n-6
PA	Palmitic acid, 16:0
SA	Stearic acid, 18:0
LA	Linoleic acid, 18:2 n-6
EPA	Eicosapentaenoic acid, 20:5 n-3
DHA	Docosahexaenoic acid, 22:6 n-3
DGLA	Dihomo-gamma-linolenic acid, 20:3 n-6
OA	Oleic acid, 18:1 n-9
VA	Vaccenic acid, 18:1 n-7
ROS	Reactive oxygen species
D5d	Delta 5 desaturase (20:4/20:3)
D6d	Delta 6 desaturase (DGLA/LA)
D9d	Delta 9 desaturase (18:1 n-9/18:0)
Elo5	Elongase 5 (VA/PA)
Elo6	Elongase 6 (18:0/16:0)
FA	Fatty acid
SFA	Saturated fatty acid
MUFA	Monounsaturated fatty acid
PUFA	Polyunsaturated fatty acid
EM	Milk-based group
EF	Fish-based group
ES	Standard group
MDA	Malondialdehyde
SOD	Superoxide dismutase
GPx	Glutathione peroxidase
CAT	Catalase
Na	Natrium
Mg	Magnesium
K	Kalium
Ca	Calcium
Cr	Chrome
Mn	Mangan
Fe	Iron
Cu	Copper
Zn	Zinc
Se	Selenium
As	Arsenic
Cd	Cadmium
Hg	Mercury
Pb	Lead
